# Welcome to *Thyroid Research*

**DOI:** 10.1186/1756-6614-1-1

**Published:** 2008-09-29

**Authors:** Andrzej Lewinski, Josef Köhrle, Maria Alevizaki, Barbara Jarzab

**Affiliations:** 1 Department of Endocrinology and Metabolic Diseases, Medical University of Lodz, Lodz, Poland; 2 Institut für Experimentelle Endokrinologie, Humboldt Universität, Berlin, Germany; 3 Endocrine Unit, Evgenidion Hospital and Department of Medical Therapeutics, Athens University School of Medicine, Athens, Greece; 4 Department of Nuclear Medicine and Endocrine Oncology, MSC Memorial Cancer Center and Institute of Oncology, Gliwice, Poland

## Abstract

Welcome to the first issue of *Thyroid Research*, a new journal published by BioMed Central, which aims at providing a platform for both researchers and clinicians to discuss a broad spectrum of thyroidology and related issues. These include physiological mechanisms of thyroid hormone action, secretory regulations, immunological and genetic aspects and, finally, news and information on state-of-the-art diagnostic equipment and treatment protocols for more effective management of thyroid disorders.

## Editorial

It is our great pleasure, as founding Editors-in-Chief of *Thyroid Research*, to participate in its launch and welcome you here with this introduction. At this point our congratulations and grateful thanks go to the Editors, members of the Editorial Board and all others involved, for their invaluable contribution to this ambitious project. To mention but a few among many, our special thanks go to Mr. Stefan Busch, Ms. Andrea Melendez and Ms. Anne Braae from BioMed Central for their continuing support in the launching of *Thyroid Research*.

Thyroidology, in common with other medical specialties, is going through an exciting dynamic phase of change, as a result of recent progress in basic sciences such as physiology, biochemistry, pharmacology, immunology and genetics. Consequentially, the main purpose of *Thyroid Research *is to provide to physicians and scientists with new methodologies, insights and guidelines leading to more effective management of thyroid diseases in the broadest sense, including their effects on other systems. Thus *Thyroid Research *will try to cover all aspects of thyroid disorders and thyroid hormone action, including their effects on metabolism and the cardiovascular, pulmonary, gastroenterological and nervous systems.

*Thyroid Research *aims to provide scientists and front-line physicians with a chance to publish their achievements pertaining to thyroidology, the fastest-growing field in endocrinology in a journal dedicated to this subject.

We would like to use the opportunity of this welcome to document the history of the founding of this journal. First, this would have never been achieved without the understanding and support expressed by many leading endocrinology experts in Poland, Europe and the rest of the world. *Thyroid Research *is officially affiliated with the Polish Society of Thyroidology and has been recognised by a number of European endocrinology institutions. In view of this initial warm welcome that *Thyroid Research *has received from top endocrinologists, it has the ambition to become a renowned thyroidology journal, highlighting the ideas and spreading the advantages of European research and clinical management in the thyroid field across the globe. Taking advantage of electronic publishing, the contents of *Thyroid Research *will be freely available via the open access model to all interested organizations and individuals. We aim at a quality publication in the service of all health professionals, including clinical specialists and basic science researchers.

This journal will be open to all points-of-view. The guiding rule will be to maintain high scientific standards. We also intend to welcome controversy and encourage debate. When appropriate, we will invite and publish peer commentaries. We welcome contributions which challenge the readership with new ideas and perspectives. We urge contributors to take advantage of the medium in new and innovative ways. We will do our best to continuously support such efforts. From this point on, we will do everything we can to streamline and expedite the publication process. Time will only tell if we are successful, but we certainly intend to try our best.

So, welcome again! We encourage you to visit the website often to stay up-to-date with the latest articles published in *Thyroid Research*. We look forward to reviewing your submissions.

